# Predicting the impact of targeted fence removal on connectivity in a migratory ecosystem

**DOI:** 10.1002/eap.3094

**Published:** 2025-01-27

**Authors:** Imogen A. Schwandner, Thomas A. Morrison, J. Grant C. Hopcraft, Jake Wall, Lacey Hughey, Randall B. Boone, Joseph O. Ogutu, Andrew F. Jakes, Shem C. Kifugo, Campaign Limo, Stephen Ndambuki Mwiu, Vasco Nyaga, Han Olff, Gordon O. Ojwang, Wilson Sairowua, Jackson Sasine, Jully S. Senteu, Daniel Sopia, Jeffrey Worden, Jared A. Stabach

**Affiliations:** ^1^ School of Biodiversity, One Health and Veterinary Medicine University of Glasgow Glasgow UK; ^2^ Smithsonian National Zoo and Conservation Biology Institute Conservation Ecology Center Front Royal Virginia USA; ^3^ Geography Department Humboldt Universität zu Berlin Berlin Germany; ^4^ Mara Elephant Project, Lemek Conservancy Kenya; ^5^ Department of Ecosystem Science and Sustainability and the Natural Resource Ecology Laboratory Colorado State University Fort Collins Colorado USA; ^6^ Biostatistics Unit, Institute of Crop Science University of Hohenheim Stuttgart Germany; ^7^ Wyoming Migration Initiative, Wyoming Cooperative Fish and Wildlife Research Unit, Dept 3166 University of Wyoming Laramie Wyoming USA; ^8^ Groningen Institute for Evolutionary Life Sciences University of Groningen Groningen The Netherlands; ^9^ Kenya Wildlife Service Nairobi Kenya; ^10^ Department of Wildlife Populations and Habitat Dynamics Wildlife Research and Training Institute Naivasha Kenya; ^11^ Pardamat Community Conservation Area Maasai Mara Wildlife Conservancies Association Narok Kenya; ^12^ Maasai Mara Wildlife Conservancies Association Narok Kenya; ^13^ WWF International Nairobi Kenya

**Keywords:** Circuitscape, connectivity, corridors, East Africa, fencing, grassland restoration, land‐use change, linear barriers, migration, pastoralism, ungulates, wildebeest

## Abstract

Fencing is one of the most widely utilized tools for reducing human‐wildlife conflict in agricultural landscapes. However, the increasing global footprint of fencing exceeds millions of kilometers and has unintended consequences for wildlife, including habitat fragmentation, movement restriction, entanglement, and mortality. Here, we present a novel and quantitative approach to prioritize fence removal within historic migratory pathways of white‐bearded wildebeest (*Connochaetes taurinus*) across Kenya's Greater Masai Mara Ecosystem. Our approach first assesses historic and contemporary landscape connectivity of wildebeest between seasonal ranges by incorporating two sets of GPS tracking data and fine‐scale fencing data. We then predict connectivity gains from simulated fence removal and evaluate the impact of different corridor widths and locations on connectivity and removal costs derived from locally implemented interventions. Within the study system, we found that modest levels of fence removal resulted in substantial connectivity gains (39%–54% improvement in connectivity for 15–140 km of fence line removed). By identifying the most suitable corridor site, we show that strategically placed narrow corridors outperform larger, more expensive interventions. Our results demonstrate how and where targeted fence removal can enhance connectivity for wildlife. Our framework can aid in identifying suitable and cost‐effective corridor restoration sites to guide decision‐makers on the removal of fences and other linear barriers. Our approach is transferable to other landscapes where the removal or modification of fences or similar barriers is a feasible mitigation strategy to restore habitat and migratory connectivity.

## INTRODUCTION

Linear barriers to movement, such as fences, roads, railways, or pipelines are restricting animal movements globally (Kauffman et al., 2021; Laurance et al., 2014; Tucker et al., 2018) and contribute to the loss of terrestrial mammal migrations worldwide (Ben‐Shahar, 1993; Harris et al., 2009; Jones et al., 2022; Nandintsetseg et al., 2019). Of these barriers, fencing is documented to have particularly deleterious effects on the proliferation of migratory mammals (Creel et al., 2013; Packer et al., 2013; Pfeifer et al., 2014; Woodroffe et al., 2014a, 2014b), including reductions in gene flow and access to resources (Jakes et al., 2018), as well as direct mortality associated with entanglement (Eacker et al., 2023; Harrington & Conover, 2010).

In response, conservationists have appealed for the removal or modification of fences in favor of “wildlife‐sensitive land‐use planning” (Woodroffe et al., 2014b), with the goal of creating landscapes that account for the needs of both people and wildlife (Kremen & Merenlender, 2018). Indeed, connectivity restoration is a stated goal of the Kunming‐Montreal Global Biodiversity Framework (Hughes, 2023).

There are opportunities to use spatial information and modeling techniques to inform the removal and modification of fence barriers and to reconcile conservation and future developments (Sawyer et al., 2009). Yet conservation outcomes following fence removal have only been documented sporadically, especially as most fence lines and their attributes have yet to be mapped. In Botswana, for instance, removing veterinary fencing has led to the re‐establishment of critical habitat connections for migrating zebra (*Equus quagga*; Bartlam‐Brooks et al., 2011). Similarly, in South Africa, de‐fencing the western border of Kruger National Park quickly resulted in elephants (*Loxodonta africana*) recolonising a neighboring game reserve after a 33‐year hiatus (Hiscocks, 1999). The historic wildebeest migration occurring to the west of the Greater Kruger Ecosystem, however, has not resumed (Owen‐Smith et al., 2020).

Here, we present a framework for identifying priority sites for fence removal, across the Greater Masai Mara Ecosystem (GMME) in Kenya. Renowned for its rich cultural history and biological diversity, the GMME has experienced rapid land‐use changes which have accelerated in the last 15 years (Løvschal et al., 2017, 2022), threatening the persistence of people and wildlife (Lankester & Davis, 2016; Mwangi, 2007; Serneels & Lambin, 2001). The transition from communal to private land ownership has driven the proliferation of fencing (Løvschal et al., 2017, 2022; Tyrrell et al., 2022) to protect and demarcate private property and maintain pastures for livestock grazing (Mwangi, 2007; Said et al., 2016; Weldemichel & Lein, 2019). By 2022, this resulted in the fencing of nearly 1/5th (1303 km^2^) of land across the GMME (Løvschal et al., 2022). These fences limit the ability of wildlife and livestock to track ephemeral sources of forage and water (Boone et al., 2006; Reid et al., 2008), contributing to precipitous population declines (Ogutu et al., 2011, 2013, 2016; Serneels & Lambin, 2001) and degradation of key ecological processes driven by migration, such as nutrient transport (Bauer & Hoye, 2014). Wildebeest, in particular, are highly mobile and yet poor at jumping fences. Given their sheer numbers, wide range, and intensive modification/regulation of the landscape, wildebeest have been considered a keystone species in the GMME (Sinclair & Byrom, 2006).

Restriction of movement negatively impacts both wildlife and pastoralist livelihoods in the region (Boone & Hobbs, 2009; Little et al., 2008; Næss, 2013) and fencing is costly to establish and maintain (Weldemichel & Lein, 2019). There is therefore urgent need for landscape‐level planning that balances the costs of both intervention and impacts on livelihoods to manage fencing and connectivity in unprotected rangelands such as the GMME (Durant et al., 2015; Xu & Huntsinger, 2022).

Despite the rapid pace of landscape change occurring in the GMME, there is considerable interest from landowners to identify interventions that improve both wildlife conservation and livelihoods. To address this need, we present an approach that combines long‐term GPS tracking data, fine‐scale spatial fence barrier data, local compensation payment figures, and Circuitscape modeling to derive predictions of connectivity improvements following different intervention scenarios for prioritizing cost‐effective solutions. Specifically, we (1) quantify changes between historic and contemporary landscape connectivity driven by fence densification; (2) validate modeled connectivity losses with observed changes in an independent validation movement dataset; and (3) simulate targeted fence removal to identify removal priorities whilst balancing the needs of people and wildlife. While our study examines the impact of fence removal on wildebeest connectivity, we highlight how our framework could be applied to a wide range of landscapes where there is a need to optimize the removal or modification of linear barriers that affect animal movement.

## MATERIALS AND METHODS

### Study area

**FIGURE 1:**
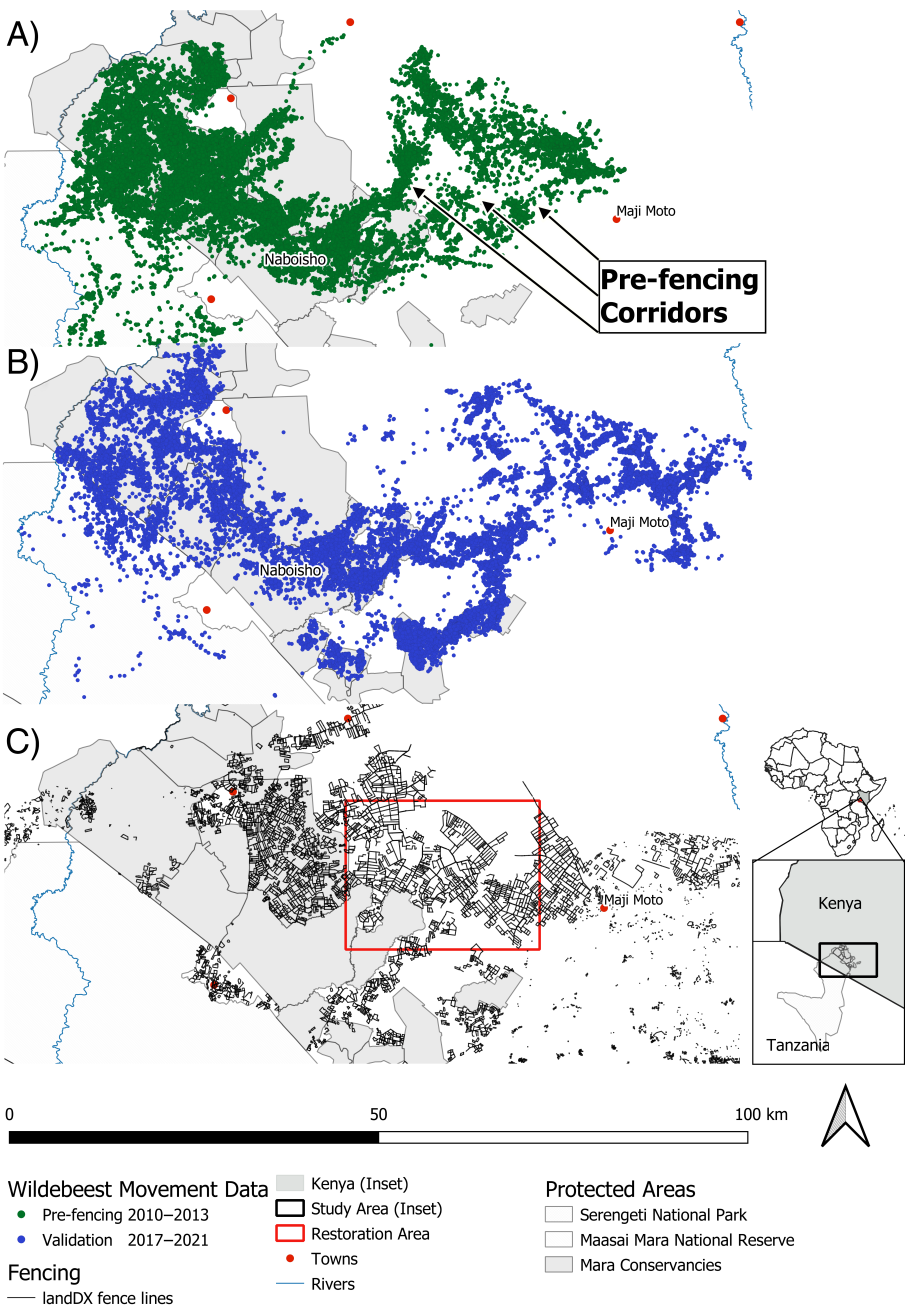
(A) Locations of pre‐fencing GPS collared wildebeest (*n* = 13 individuals, 2010–2013) corresponding to a period before fencing expansion. Broadscale movements were observed between the Mara Plains and the Loita Plains with three clear main migratory corridors in the East. (B) Locations of validation data GPS collared wildebeest (*n* = 9 individuals, 2017–2021) during a period of extensive fence densification (fencing period), in areas directly adjacent to private and community conservancies. (C) Fencing extent in 2022, based on the landDX database (Tyrrell et al., 2022). Restoration area of interest shown in red.

The GMME is located in Narok County, southwestern Kenya (1°15′ S, 35°20′ E) and borders the Serengeti National Park, Tanzania, to the south (Figure 1). The region is characterized by semi‐arid and arid grassland savannas and high interannual variability in rainfall with multiple geographical gradients (Bartzke et al., 2018; Ogutu et al., 2011). These gradients underlie the movements of diverse assemblages of wild and domestic large mammals which range across a matrix of protected areas, conservancies, and community lands. Løvschal et al. (2017) describe land management in the region and how it relates to fencing whereas Sinclair et al. (2008) provide a detailed ecological description of the study system. The Mara‐Loita wildebeest population migrates predominantly in east–west directions from the Mara reserve and mixes with the larger Serengeti‐Mara migratory population during the dry season (July–October). While the majority of the Serengeti‐Mara migration lies within protected area boundaries, the Mara‐Loita migration occurs predominantly in unprotected rangelands (Stabach et al., 2022) where the anthropogenic footprint has expanded rapidly. The Mara‐Loita population has declined from over 109,000 animals in 1977–1978 to under 27,000 animals in 2022 (see Appendix S1: Figure S1; G. O. Ojwang et al., unpublished data).

### Data

#### Movement data

Wildebeest GPS tracking data were collected from unique individuals over two periods: (1) a historic “pre‐fencing” period (2010–2013) that preceded the rapid densification of fencing across the region, and (2) a “fencing” period (2017–2021) coincident with fence expansion (Løvschal et al., 2022). We use the term “pre‐fencing” for convenience, since fencing across the ecosystem actually began in the 1950s. The amount, density, and spread of fencing, however, remained relatively low and stable until dramatic changes occurred post‐2013 (Løvschal et al., 2022). All aspects of animal handling were conducted under the direction of a Kenya Wildlife Service or Tanzania Wildlife Research Institute field veterinarian and approved by the International Animal Care and Use Committees at Colorado State University (Approval No. 09‐214A‐02) and the Tanzania Commission for Science and Technology (Permit No. 2021‐33‐NA‐2007‐034). Captured animals were selected at random from spatially disparate areas and, presumably, distinct social groups.

##### Pre‐fencing data (2010–2013)

All pre‐fencing movement data are available on Movebank (Stabach et al., 2020), with a detailed summary of individual movements provided in Stabach et al. (2022). Our analyses incorporated 13 wildebeest (5 male; 8 female) fitted with Lotek WildCell GPS Collars that moved exclusively on the Kenyan side of the ecosystem between the Mara and Loita Plains (Figure 1A). Animals were tracked between 16 and 964 days (median = 538 days). Data were standardized to a 3 h collection interval.

##### Validation data (2017–2021)

Ten female wildebeest were fitted with Followit GPS collars between 2017 and 2021. Animals were tracked between 30 and 1692 days (median = 960 days), with GPS fixes collected every 1–6 h. Data were downsampled to 4 h intervals for analysis. One individual (animal W69) was excluded from analysis as it migrated south to the Serengeti. These GPS data were used to validate (1) the habitat suitability model, (2) predictions of connectivity based on models fit to pre‐fencing movement data, (3) predictions of connectivity incorporating fencing, and (4) the model of connectivity change between the historic pre‐fencing and fenced connectivity scenario. Validation was performed at two scales: (1) the regional scale across the entire Mara and (2) the local scale covering only the restoration area of interest (see Figure 5C).

#### Fencing data

Fence polylines were collated from several sources and either digitized from satellite imagery or collected via the TerraChart Android application (https://play.google.com/store/apps/details?id=org.maraelephantproject.terrachart) by trained field staff of the Mara Elephant Project (2019–2022) before being uploaded to the landDX database (Tyrrell et al., 2022; downloaded May 2022). LandDX is an open‐access, fine‐scale land‐cover geospatial dataset covering southern Kenya. Dynamic updates to the data portal (https://www.arcgis.com/home/search.html?q=landDx) are made monthly.

### Connectivity modeling

**FIGURE 2:**
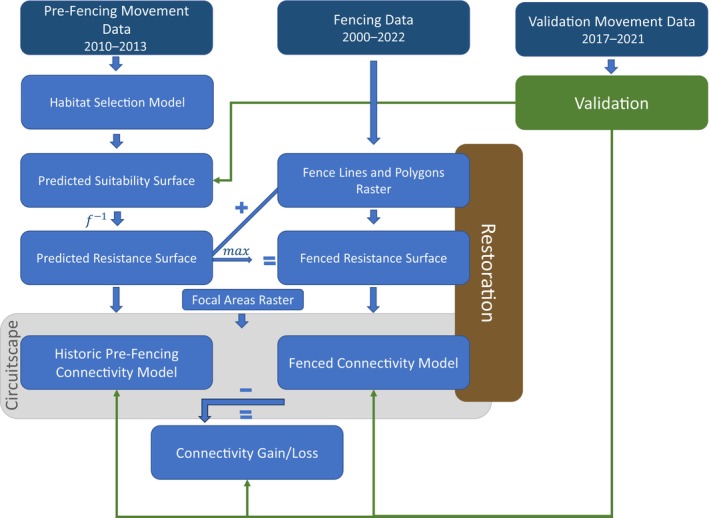
Graphical summary of the analytical approach. Datasets (top) highlighted in dark blue, intermediate and final outcomes in light blue, and Circuitscape analyses of connectivity in light gray. Layers altered for connectivity restoration modeling are highlighted in brown. Step one was to predict habitat suitability using a habitat selection model based on historic movement data. Using the inverse of suitability as a resistance surface, historic pre‐fencing (2010–2013) and fenced (2022) connectivity levels between specified focal areas were then predicted in Circuitscape either including (fenced) or excluding (pre‐fencing) fencing in the resistance surface. Validation movement data (2017–2021) were used to validate predicted outcomes (green arrows). Finally, we assessed the impact of simulated fence removal on connectivity restoration.

Figure 2 summarizes our general workflow. All analyses were conducted in R (v. 4.2.0; R Core Team, 2022). Further details are provided below.

#### Historic connectivity levels (2010–2013)

We developed a habitat selection model (following Stabach et al., 2016) to predict habitat suitability across the area. Habitat selection was based on the pre‐fencing movement data. Around each occurrence point, 50 pseudo‐absence points were generated and randomly distributed within a buffer of the individual maximum observed step‐length of each animal (step length group sample mean: 11 km and SD: 9 km). Independent variables included in habitat selection analyses were NASA's MODIS 16‐day Normalised Diference Vegetation Index (NDVI) product (MOD13Q1) interpolated between the two nearest 16‐day NDVI values for that location, rate of change in NDVI (i.e., ΔNDVI, difference between nearest 16‐day NDVI values), topographic wetness index (TWI), anthropogenic footprint (distance to human settlements weighted by population density; Stabach et al., 2016), and the distance to woody vegetation, rivers, primary and secondary roads (see Appendix S1: Table S1 for more details). All data layers were resampled to 50 m^2^ resolution using bilinear interpolation (cf. Stabach et al., 2016), to capture the detail of the fencing and habitat covariate data, within a reasonable computing time. Time‐varying covariates (i.e., NDVI, ΔNDVI) were matched to each GPS position's time‐stamp. For all distances, a quadratic term was included to account for expected non‐linear biological responses (Hopcraft et al., 2014; Stabach et al., 2016). Occurrence/pseudo‐absences were fit to a generalized linear mixed model (GLMM) with a logit link function and a binomial error distribution using the lme4 package (Bates et al., 2015), after confirming that model assumptions regarding homoscedasticity and normality of residuals were met using the “DHARMa” package (Hartig & Lohse, 2022). We assessed multi‐model inference with the MuMIn package (Bartoń, 2022) using an information theoretic approach (Burnham & Anderson, 2002).

Habitat suitability was then predicted across the study area using the most parsimonious resource selection model. For this prediction, we set NDVI to its long‐term mean (2000–2022). Because the goal of our analysis was to capture suitability during the migratory phase when wildebeest would have moved through the fenced area, we set ΔNDVI to an early wet‐season period (March 2011; green‐up period) when ΔNDVI values were relatively homogenous across the landscape. The inverse of predicted habitat suitability was then used for the resistance surface in connectivity modeling (Crego et al., 2021; Osipova et al., 2018).

Historic pre‐fencing and fenced connectivity levels across the Mara were characterized using Circuitscape (v. 4.0; McRae et al., 2008) using the resistance surface and three focal areas of interest in pairwise‐mode, connecting each cell to its four nearest neighbors. Circuitscape uses conductance theory to model connectivity as a current of animals between user‐specified focal nodes across a rasterized resistance surface. Outputs show the cumulative current (of animals) across each cell incorporating all possible pathways through the landscape. Setting a threshold of a cumulative current >0.003 (top 10% of pixels in the connectivity model), we identified historic corridors in the model.

**FIGURE 3:**
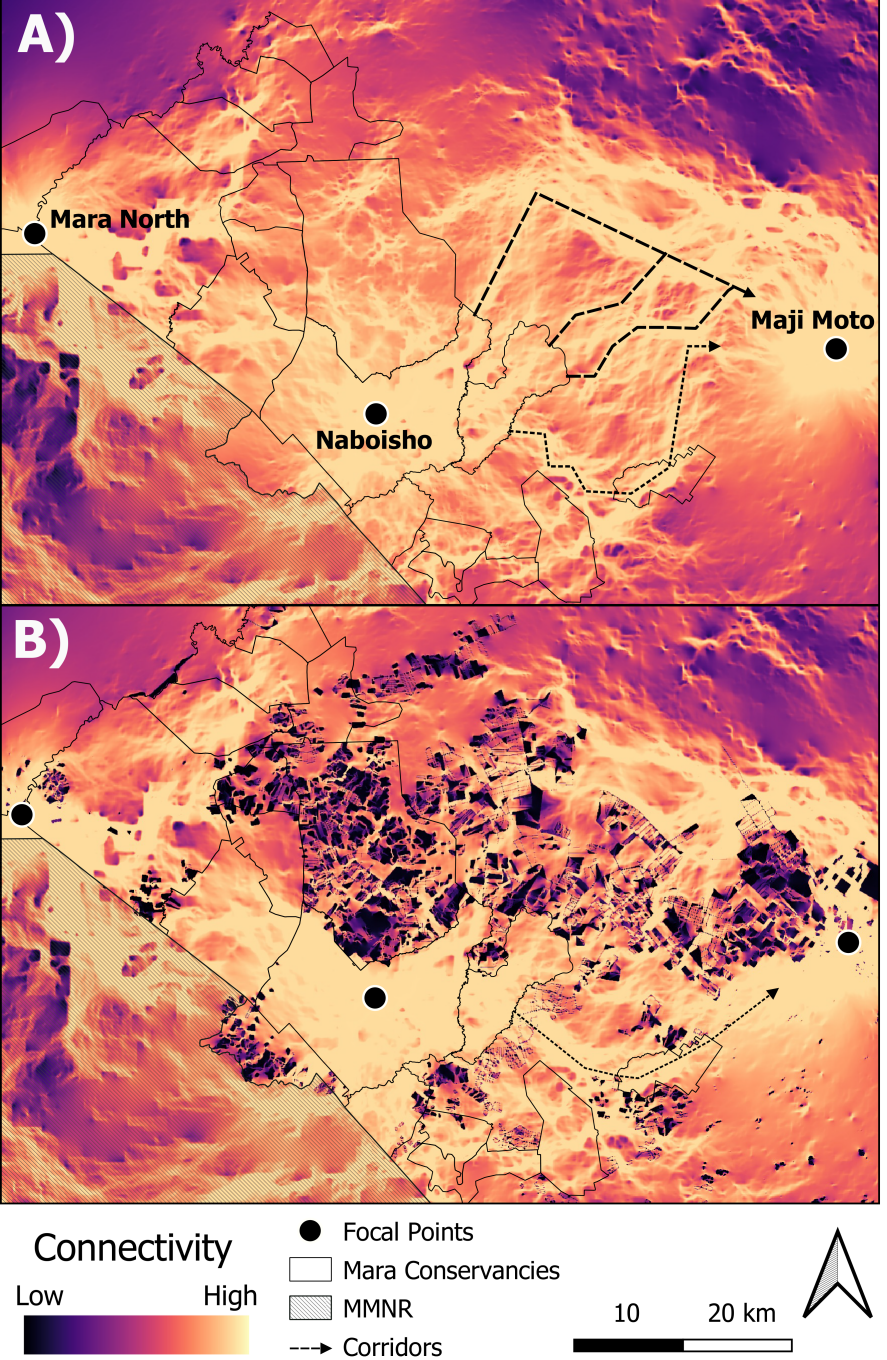
(A) Historic pre‐fencing (2010–2013) and (B) fenced (2022) connectivity levels for wildebeest across the Greater Masai Mara Ecosystem, based on Circuitscape models. Connectivity was measured as the cumulative current between three focal points: Maji Moto, a wet season area, and the Naboisho and Mara North conservancies, both dry season areas for wildebeest adjacent to the Masai Mara National Reserve (MMNR). Several corridors between Naboisho and Maji Moto are apparent and depicted as dashed (historically used corridors) and dotted (only modeled corridors) lines. All corridors north of the Naboisho conservancy have been lost or significantly altered following recent fencing (2022), with connectivity diverted through a single southern corridor (dotted line in B). We did not observe wildebeest using this route during the historic pre‐fencing tracking period (2010–2013). These patterns can already partially be observed in Figure 1.

Three focal areas provided end nodes for the connectivity analysis: (1) the Loita Plains (wet‐season range), near Maji Moto village—the most eastern extent of observed historic movements of the collared animals; (2) the centroid of the Naboisho conservancy—an important stepping‐stone on the migratory route; and (3) in the Mara Plains (dry season range), where the Mara River bounds Mara North conservancy and partially restricts westward movement of collared individuals (see Figure 3).

To validate the accuracy of the historic pre‐fencing habitat suitability model, the pre‐fencing and fenced connectivity model, and the connectivity change we used the 2017–2021 GPS movement dataset, collected during a period of rapid fence expansion (Løvschal et al., 2022). We generated 50 pseudo‐absences per occurrence from the validation data, similar to above (i.e., within a buffer of maximum individual step length in 4‐h interval; group sample: 17.3 ± 8.8 km [mean ± SD]) to model whether validated metrics (predictions of historic pre‐fence habitat suitability, historic pre‐fence and fenced connectivity, connectivity change) significantly differed between presences and pseudo‐absences. Validation of each metric was performed at the regional scale (across the entire Mara) and at a finer scale (covering only the restoration area of interest, see Figure 5C). If the historic pre‐fencing models accurately predicted habitat suitability and connectivity of wildebeest, we expected habitat suitability and connectivity to be higher at occurrence locations when compared with pseudo‐absences generated from the validation dataset at both scales. However, we expected weaker effects for connectivity at the local scale. We assumed that although the environment had changed between time periods, wildebeest selection at either scale remained the same. In addition, while we expected contemporary wildebeest (the validation data) to closely track pre‐fencing connectivity across the region, we also recognized that animals might have been unable to do so at the local restoration scale because of existing fencing—blocking access to previous high connectivity areas.

At the regional scale, we fitted a GLMM with a beta error distribution and a logit link function (glmmTMB; Brooks et al., 2017) to assess the difference in habitat suitability and a linear mixed model of log transformed current (connectivity) values in the lme4 package (Bates et al., 2015) to assess the difference in habitat connectivity between presences and pseudo‐absences. At the local scale, we transformed suitability (logit) and pre‐fencing connectivity (log) to assume a normal distribution to model with the lme4 package (Bates et al., 2015). We checked model assumptions using the DHARMa package (Hartig & Lohse, 2022). Additional details on the models are provided in the Appendix S1: Section S1.

#### Fenced connectivity levels (2022) and connectivity change

Existing fencing (as of May 2022), as well as parcels completely enclosed by fencing, were incorporated into the resistance surface by assigning a resistance value of 100 (i.e., no movement; McRae et al., 2012; Osipova et al., 2018). While wildebeest can jump certain fences, they are recognized as poor jumpers and often become entangled in fences. Data on specific responses to different fence types or heights, however, is lacking. We therefore treated all fences (regardless of type) as equally resistant. Our model, hence, represents a worst‐case scenario, with permeability possibly higher in some areas, depending on fence type, height, and density.

**FIGURE 4:**
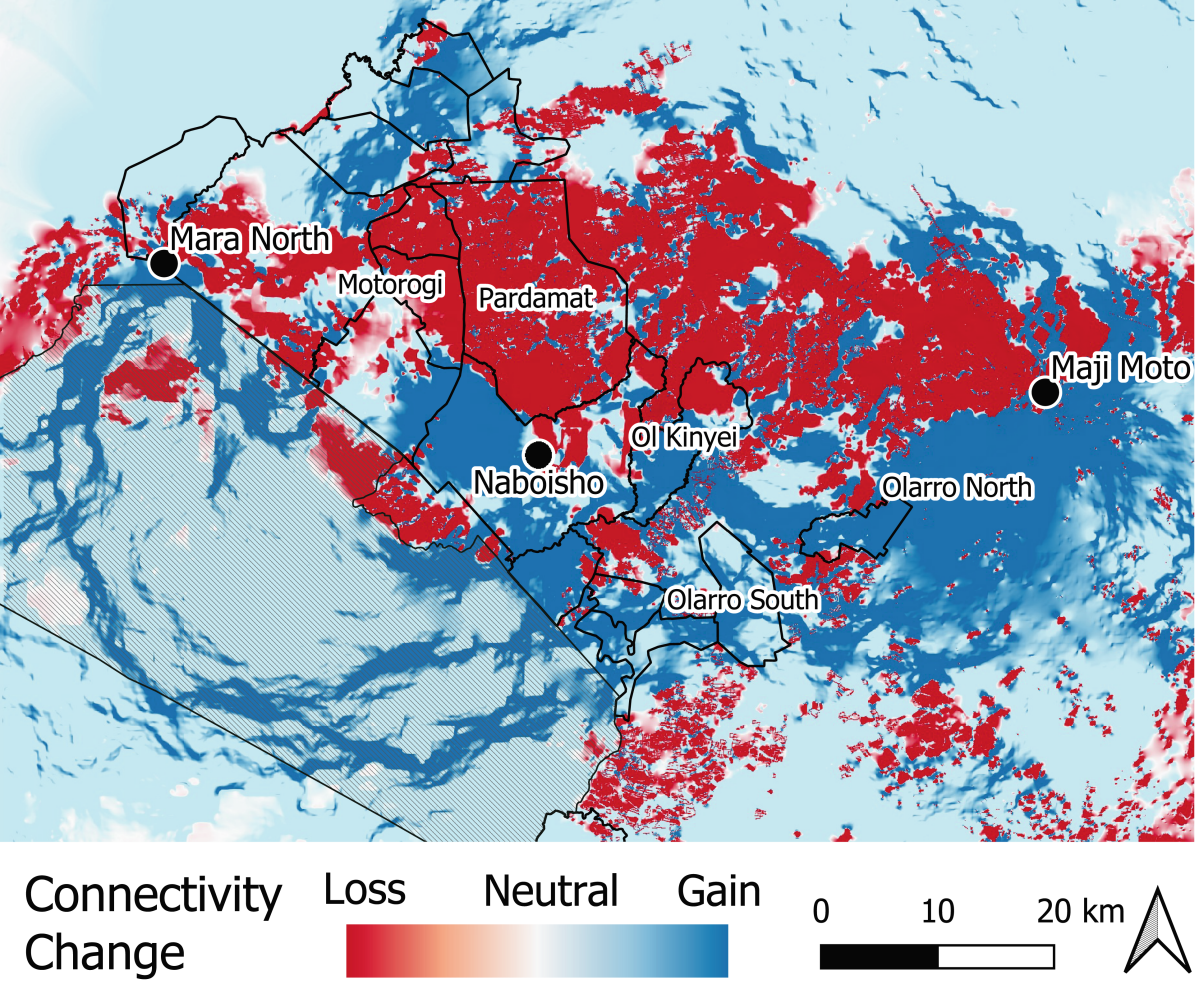
Predicted change in wildebeest connectivity due to fencing between fenced (2022) and historic pre‐fencing (2010–2013) levels across the Greater Masai Mara Ecosystem, Kenya, based on Circuitscape models. Focal nodes of connectivity analysis appear as black dots with labels. Other labels show conservancies marking notable gains or losses.

We validated the fenced connectivity model (high fence density) and the difference between the current and historic levels of connectivity (calculated by subtracting modeled connectivity in 2022 by modeled connectivity from 2010 to 2013; Figure 4), by fitting linear mixed models using the lme4 package (Bates et al., 2015) to assess metrics at both spatial scales. Fenced connectivity was log transformed. We checked model assumptions using the DHARMa package (Hartig & Lohse, 2022). Additional details are provided in Appendix S1: Section S1.

We expected wildebeest GPS locations (2017–2021) to commonly occur in areas of connectivity gain but not loss. This would result in the mean current difference to be higher in random locations within the predefined area and indicate that the connectivity map, including fencing (2022), was accurate at the local scale. We expected the opposite at the regional scale, where connectivity loss is more prominent than connectivity gain. Although fencing continued to increase after the validation data were collected (2017–2021), animals would have been impacted by sufficient fencing to justify validating the changes observed in the 2022 landscape. We expected wildebeest to occur in areas of higher levels of connectivity at the regional scale, but that this relationship would be weaker in the restoration area at the local scale.

#### Simulated fence removal to restore connectivity and removal costs

**FIGURE 5:**
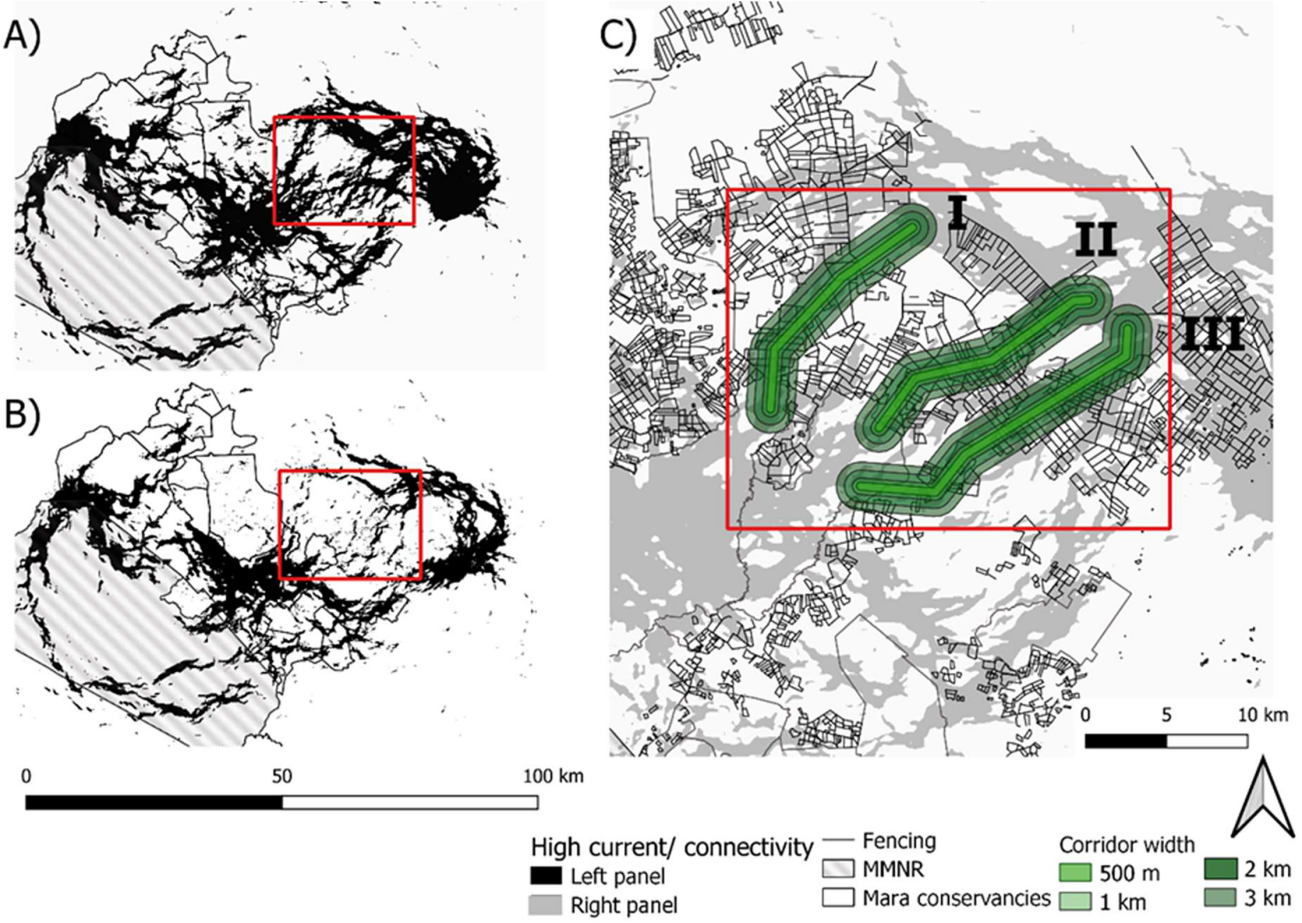
Restoration corridor scenarios for fence removal. High‐current/connectivity areas (>0.003) during (A) pre‐fencing and (B) fenced period, based on Circuitscape models (Figure 3) and (C) three candidate routes connecting dry season ranges (Naboisho and Ol Kinyei conservancies) with the wet season range (Loita Plains). This particular area was chosen as a plausible site for simulated restoration because of observations of empirical movement data, historically high connectivity, and relatively low levels of fencing. Corridor widths of 0.5, 1, 2 and 3 km are illustrated for each of the three candidate routes. MMNR ‐ Masai Mara National Reserve.

In areas that showed high connectivity loss, three historic (pre‐fencing) corridors were targeted to remove fences from the resistance surface to assess the extent by which fence removal restored connectivity (see Figure 5). All three candidate corridors were located northeast of Naboisho/Ol Kinyei conservancies based on (1) initial observations of the raw movement data where these habitat connections were clearly visible (see Figure 1A), (2) where values of high connectivity (>0.003) were identified in the pre‐fencing connectivity model (Figure 5A), and (3) the raw fencing data. We did not consider the 4th southern corridor (Figure 3A dotted line) for restoration as pre‐fencing wildebeest never used it and the route through very densely fenced areas would have required far too extensive (and hence costly) fence removal. Each corridor was digitized in QGIS v.3.16 (QGIS Development Team, 2022). We focused on this area to compare the impact of fence removal on functionally viable routes that also hold promise for restoration because of relatively short distances between unfenced grazing habitats. A key priority was minimizing fence removal effort for each route while closely tracking pre‐fencing high connectivity areas.

We examined corridor widths of 0.5, 1, 2, and 3 km for each corridor location (totalling 12 scenarios) and measured the improvement in connectivity for each corridor against the cost of fence removal based on previously paid compensation in USD (proportional to the total area covered by fences removed). To assess improvement, cumulative current was summed across a region of interest that covered all three corridors for each restoration scenario. Improvement was measured as the proportion of connectivity loss that could be restored.

To estimate removal costs for each corridor site and width, all fenced land parcels were digitized from the fence line data using a polygonising algorithm and additional manual digitisation in QGIS v.3.16. Land parcels were considered “fenced” if they (1) were enclosed by at least three fence lines—of those two at least parallel or angled toward another or (2) lied fully within a larger fully enclosed parcel extending outside the corridor. The total fenced area within each corridor scenario was then quantified to estimate the cost of intervention at each site for each scenario, based on fence removal cost estimates from Pardamat Conservation Area (PCA) where landowners received a once‐off payment of 10.000 KES or approximately US$75 (April 2023) per acre to permanently de‐fence their land.

## RESULTS

### Historic pre‐fencing connectivity (2010–2013)

The habitat selection model using pre‐fencing data estimated selection coefficients that were close, in terms of size and direction of effect, to those reported in Stabach et al. (2016) (see Appendix S1: Table S5). Results from a validation test (summarized in Table 1) showed that habitat suitability values were higher at occurrence locations in the 2017–2021 data than at pseudo‐absence locations at both the regional and local scales (Regional—β_Presence_ = 0.55 ± 0.006; Local—β_Presence_ = 0.44 ± 0.02). The pre‐fencing connectivity model identified three main corridors—continuous high connectivity (>0.003) pathways, leading from the Naboisho conservancy to the Loita Plains (dashed lines in Figure 3A; Figure 5A). These closely track the already identified corridors from the raw movement data (see Figure 1A). Validation with the 2017–2021 movement data showed occurrence locations consistently in areas with higher current than random locations regionally and locally (Regional—β_Connectivity_ = 0.47 ± 0.005; Local—β_Connectivity_ = 0.078 ± 0.008). The model highlighted a fourth corridor from the Naboisho/Ol Kinyei conservancies through Olarro North conservancy toward the Loita Plains (Figure 3A dotted line; Figure 5A). Interestingly, wildebeest from our historic pre‐fencing dataset (2010–2013) never used this connection (Figure 1A), but it is closely tracked by wildebeest from our validation dataset (Figure 1B).

**TABLE 1:** Validation results at the regional and local scales.

Model	Regional scale (entire Mara)		Local scale (restoration area)	
	Coefficient	SE	Coefficient	SE
Habitat suitability	0.55	0.0057	0.44	0.02
Pre‐fencing connectivity	0.47	0.0048	0.078	0.008
Fenced connectivity	0.37	0.007	0.175	0.021
Connectivity difference	0.00024	0.00001	−0.00072	0.00003

*Note*: Coefficients and SEs of generalized linear mixed models of the effect of presence/pseudo‐absence on different modeled metrics. All coefficients are significant at the 0.01 level.

### Fenced connectivity levels

The connectivity model, including all mapped fences (in 2022), clearly showed degradation of the northern corridors and circumvention of the main fenced areas in favor of a stronger southern corridor that deviates from the pre‐fencing fourth corridor (Figures 3A,B and 5A,B). Figure 4 highlights areas of connectivity gain and loss across the study area. Losses in connectivity have predominantly occurred in the Motorogi conservancy (including unprotected land northward), the PCA, and the western adjoining unprotected rangelands.

Wildebeest, impacted partially by fencing (validation movement data from 2017 to 2021) moved in areas where we predicted connectivity gain, rather than loss (due to fencing), at the regional scale (Regional—β_ConnectivityChange_ = 0.00024 ± 0.00001). In other words, our model predicted the altered movement circumventing fenced areas with high connectivity loss well. At the local scale (restoration area of interest) where connectivity is predominantly lost and only very few areas exhibit gains, wildebeest moved in areas of net connectivity loss (Local—β_ConnectivityChange_ = −0.00072 ± 0.00003). Estimation of the average distance traveled via their main routes between the Naboisho conservancy and Maji Moto showed that wildebeest from 2017 to 2021 moved about 1.5 times the distance that pre‐fencing wildebeest moved via their new diverted routes.

Validation of the fenced connectivity model showed tracking of high connectivity areas across the region (Regional—β_Connectivity_ = 0.37 ± 0.007). In other words, avoidance of fencing via use of the new predicted connective pathways. However, at the local scale where connectivity was mostly lost in the area, this relationship was much weaker (Local—β_Connectivity_ = 0.175 ± 0.021).

### Connectivity restoration and removal cost

**FIGURE 6:**
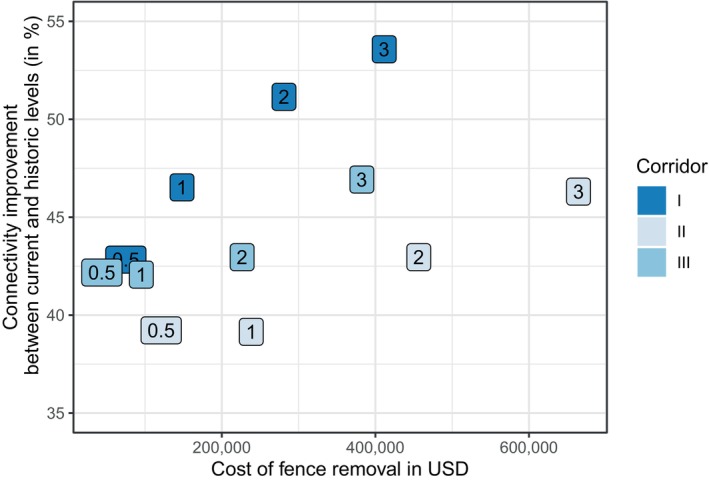
Connectivity improvement (i.e., change in cumulative “current”) measured as percentage difference in connectivity between the historic (2010–2013) and fenced (2022) levels restored through fence removal as a function of estimated removal costs in US$ along three possible corridor locations (Corridors I, II, III) with four possible corridor widths each (0.5, 1, 2 and 3 km; see marker label). Costs based on the area of land parcels enclosed by fencing intercepting the corridor. Per acre of land approximately US$75 (10.000 KES) are paid (cost from Pardamat Conservation Area).

All fence removal scenarios substantially improved connectivity (39%–54%). Of the three corridors tested, corridor I (CI; origin Naboisho) consistently achieved the highest improvements in connectivity. Corridor III (CIII) was consistently the cheapest. Corridor II was inferior to CI and CIII in both regards. Figure 6 shows connectivity improvement for each restoration scenario as a function of the cost (in USD) of de‐fencing all fenced parcels within the corridor area, where improvement was measured as the percentage of the connectivity loss between the fenced and historic levels of connectivity restored by the corridor. In general, improvements in connectivity equated to increased levels of fence removal (i.e., widths of corridors), which is proportional to cost within, but not between, corridors. For example, a 1 km wide corridor at CI was as effective as a 3 km wide corridor at location CIII, at <40% the cost.

## DISCUSSION

The fragmentation of landscapes shared by people and wildlife presents a global conservation challenge (Kauffman et al., 2021; Kremen & Merenlender, 2018; Tucker et al., 2018; Woodroffe et al., 2014b). While measures to remove or modify barriers and combat fragmentation, such as fence gaps, fence removal, or wildlife underpasses/bridges are attractive ecologically, strategies which minimize restoration costs while promoting human‐wildlife‐coexistence are essential (Xu & Huntsinger, 2022). We aimed to advance such efforts using animal movement data and predictive models to illustrate how and where fencing diminishes connectivity for migratory wildebeest and formulate a framework for prioritizing movement corridors. Individual species and species assemblage connectivity models in a nearby ecosystem show high qualitative agreement (Crego et al., 2021), suggesting that our results for wildebeest could be transferable to other migratory ungulates for which fencing impedes connectivity (Bartlam‐Brooks et al., 2011; Hiscocks, 1999). Furthermore, Jones et al. (2020) show how fence modifications in the Northern Great Plains, USA, can simultaneously benefit three sympatric ungulate species. Thus, restoration measures plausibly have positive impacts on movements of more than any single target species.

We showed that corridor creation along historic migratory routes can significantly improve connectivity. Even narrow corridors (500 m or 1 km) yielded substantial improvements to connectivity (>40% improvement). While trade‐offs remain between maximizing connectivity and minimizing removal costs, one of the study corridors (Corridor I) appears to be the most practical option among those considered to combat fragmentation and maintain connectivity in this region. For instance, Corridor I terminates in vast open areas with no fences, while Corridor III (the cheapest corridor option) terminates near much more densely fenced areas, likely requiring further remediation (Appendix S1: Figure S2). Additionally, and although covering less fenced area than Corridor I, Corridor II intersects at least twice as many land parcels as Corridor I in every scenario, requiring agreement from far more landowners to be successful. All Corridor III scenarios require removal of about twice as many individual fences, suggesting a more labour‐intensive and costly solution. Considering these findings, we encourage policymakers, government officials, and conservation practitioners to target Corridor I for immediate fence removal to maintain historic migratory connections.

There are several options to implement interventions for connectivity restoration. Løvschal et al. (2022) show that community conservancy strategies in the region can be effective at preserving fence‐free land parcels, providing additional income to pastoralists and buffering unpredictable climate change impacts. However, these interventions often exclude landowners from grazing cattle on conservancy land—one of the main drivers of conflict (Bedelian & Ogutu, 2017) and a key reason for increased illegal livestock grazing in the neighboring Masai Mara National Reserve. A non‐exclusive approach might be more suitable for establishing the proposed corridor. Payments will be needed for dismantling fences, with sufficient incentive to compensate establishment cost, as well as the foregone opportunity costs (Smallhorn‐West & Pressey, 2022) of sharing the land as a wildlife passage. However, since a primary conservation goal is to re‐establish connectivity via a relatively narrow corridor instead of securing more habitat for wildlife, landowners will not be excluded from grazing pastures, especially during droughts—a main concern of participants surveyed by Bedelian and Ogutu (2017).

Naturally, land tenure, fence type, and other socio‐economic factors will affect site remediation cost, feasibility and effectiveness, but could not be considered here due to lack of adequate data. Future studies might supplement our approach by performing a full optimization analysis that incorporates social and ecological values (Carter et al., 2020; Sage et al., 2022). Such additional constraints on fence removal could result in movement corridors being less linear than those modeled here. Spatial organization in payments and agglomeration bonuses might aid in encouraging landowner uptake and ensuring functional connectivity of corridors (Nguyen et al., 2022). We acknowledge that while the response to fences, fence gaps, and other modifications has been tested for many ungulates (Hering et al., 2022; Jones et al., 2020; Sawyer et al., 2009), including in East Africa (Wilkinson et al., 2021), the use of corridors by wildebeest is yet to be established as a prerequisite to moving forward with remediation measures.

Based on the costs of de‐fencing initiatives led by the PCA, we were able to estimate costs for each corridor intervention. We were limited to area‐, rather than, linear‐based measures of reimbursement, however, likely overestimating true costs even though we only accounted for the land portion that intersects each corridor. When delineating corridors, fence distribution limited tracking of high current areas and historic movement, as minimal fence removal was a key priority. Hence, not all corridors were modeled with equal length and direction, factors affecting corridor area and cost for remediation. A final caveat is that the single southern corridor (Figure 3B) from the fenced connectivity model deviates from the fourth corridor (Figure 3A dotted line) in the pre‐fencing model also used by 2017–2021 wildebeest (validation data) due to highly fenced areas blocking that corridor. Current in Circuitscape has to reach its destination resulting in a single southern route diverted by fencing through suboptimal (high elevation) areas unlikely to be used by wildebeest.

Many landscapes where fencing has curtailed wildlife connectivity lack detailed information on the locations of fences and on the movements of animals prior to fencing. Nonetheless, our framework could be applied to such landscapes. In open or unforested landscapes, fence locations can be predicted across space and time from aerial or satellite imagery based on strong transitions in vegetation cover (e.g., Google Microsoft Open Buildings layer; Buzzard et al., 2022; Løvschal et al., 2017). Given the rapid expansion of telemetry technologies (Rutz & Hays, 2009), proliferation of multi‐population cross‐taxa repositories of telemetry datasets (Kays et al., 2022) and efforts to improve the transferability of species distribution models across landscapes (e.g., Yates et al., 2018), predictions of wildlife connectivity in under‐studied landscapes are becoming increasingly feasible and accurate. Traditional ecological knowledge (Huntington, 2000) is another useful method to derive information on wildlife movements (Broekhuis et al., 2022). There will undoubtedly be settings or species where predictions of fencing and habitat suitability are not possible, and in these cases qualitative approaches using expert or landowner opinion may be necessary.

There are likely additional areas to the identified corridors where fence removal would be necessary to fully restore migratory connectivity for wildebeest in the Loita Plains (see Appendix S1: Figure S2). While animals can, and currently do, circumvent some of these fenced areas, removing additional fences would allow wildebeest to follow historic routes through highly suitable and well‐connected habitat. Unsurprisingly, fences are often established in areas of high suitability for wildebeest, due to the overlap in resource requirements with cattle.

Fragmentation and restricted mobility, however, threaten not only wildlife but also pastoralism which has evolved, much like migration, to allow flexibility and adaptation in variable dry‐land ecosystems (Homewood, 2008). Mobility is key to protecting pastoralist livelihoods against climate change and variability, with nomadic strategies buffering livelihoods better than sedentary strategies during climatic extremes (Little et al., 2008; Næss, 2013). Further, evidence shows that fencing reduces herbivore stocking rates (Boone & Hobbs, 2009). This, in combination with the high cost of establishing and maintaining fencing (Weldemichel & Lein, 2019), underlines the urgent need for alternative solutions in the region.

The Pardamat Conservancy Area has been leading efforts across the region to remove fencing. Løvschal et al. (2022), for example, report over 347 km of fencing removed to date. These efforts, however, are hampered by lack of coordination with neighboring conservancies, leading to isolated pockets of open land within the broader matrix of regional fencing, limiting functional connectivity. Our analysis provides an ecosystem‐wide perspective that could be integrated directly into regional land use plans, prioritizing areas with the greatest conservation impact. The recently published Narok County Physical and Land Use Development Plan (2023–2032) and the Greater Maasai Mara Ecosystem Management Plan (2023–2032) both state a well‐defined need to restore degraded areas and conserve wildebeest and other migratory species' pathways, setting the legal context to realize connectivity restoration interventions, as presented here. Such planning initiatives/efforts increasingly rely on more readily available long‐term movement data and high‐resolution mapping of anthropogenic barriers (such as the landDX database). Our analysis framework can provide a critical link between both.

Tackling complex socio‐ecological issues, such as the proliferation of fencing (Xu & Huntsinger, 2022), requires quantitative techniques, the incorporation of social sciences to inform conservation strategies, and extensive engagement with stakeholders to modify behavior and land‐use plans. Our study is a first step toward this more ambitious goal. Such strategies must recognize that while protected areas are essential for conserving biodiversity (Watson et al., 2014), they are often too small (Thirgood et al., 2004) to protect the long‐range movement needs of many terrestrial species across the globe. Hence, unprotected mixed‐use landscapes will remain crucial for biodiversity conservation (Tack et al., 2019). As we move away from land‐sparing toward land‐sharing approaches, the future of conservation will increasingly lie outside of protected areas in schemes and policies that allow for co‐existence and equitable sharing of wildlife burdens and benefits.

## AUTHOR CONTRIBUTIONS

Jared A. Stabach, Thomas A. Morrison, Imogen A. Schwandner, Grant C. Hopcraft, Lacey Hughey and Jake Wall conceived the ideas and designed methodology. Jared A. Stabach, Thomas A. Morrison, Grant C. Hopcraft, Campaign Limo, Vasco Nyaga, Stephen Ndambuki Mwiu, Jackson Sasine, Jake Wall and Wilson Sairowua collected the movement data and/or were responsible for fencing data collection. Imogen A. Schwandner, Jared A. Stabach and Thomas A. Morrison analyzed the data and led the writing of the manuscript. All authors contributed critically to the manuscript drafts and gave final approval for publication. This research is the result of close collaboration of an international research team, including researchers and practitioners in or from the study region, all of whom were engaged early on in study design and throughout the process ensuring the representation of diverse perspectives. Whenever relevant, literature published by scientists from the region was cited.

## CONFLICT OF INTEREST STATEMENT

The authors declare no conflicts of interest.

## Supporting information


Appendix S1.

## Data Availability

The fencing data were retrieved from the landDX database: https://www.arcgis.com using the query “landDX” in the search bar and downloading the file “landDx_export_active_public_uncategorized_shp.” The historic pre‐fencing wildebeest movement data (Stabach et al., 2020) are available on Movebank: https://doi.org/10.5441/001/1.h0t27719. Contemporary movement data used for validation are available on Movebank: https://www.movebank.org/cms/webapp?gwt_fragment=page%3Dstudies%2Cpath%3Dstudy4901146318. All code and data (Schwandner, 2025) are available on Zenodo: https://doi.org/10.5281/zenodo.14599735.
