# Supplementary material for: Predicting the impact of targeted fence removal on connectivity in a migratory ecosystem
Source: Ecol Appl. 2025 Jan 27;35(1):e3094. doi: 10.1002/eap.3094 (PMC11771689; doi:10.1002/eap.3094)
Supplement: Supplementary file 1 — Appendix S1. [file EAP-35-e3094-s001.pdf]

Appendix 1

of

Predicting the impact of targeted fence removal on connectivity in a migratory ecosystem

by

Imogen A. Schwandner (Corresponding author: imogenschwandner@gmail.com), Thomas A. Morrison,, J. Grant C. Hopcraft, Jake Wall, Lacey Hughey, Randall B. Boone, Joseph O. Ogutu, Andrew F. Jakes, Shem Kifugo, Campaign Limo, Stephen Ndambuki Mwiu, Vasco Nyaga, Han Olff, Gordon Ojwang, Wilson Sairowua, Jackson Sasine, Jully Senteu, Daniel Sopia, Jeffrey Worden, Jared A. Stabach

Article in Ecological Applications

Figure S1

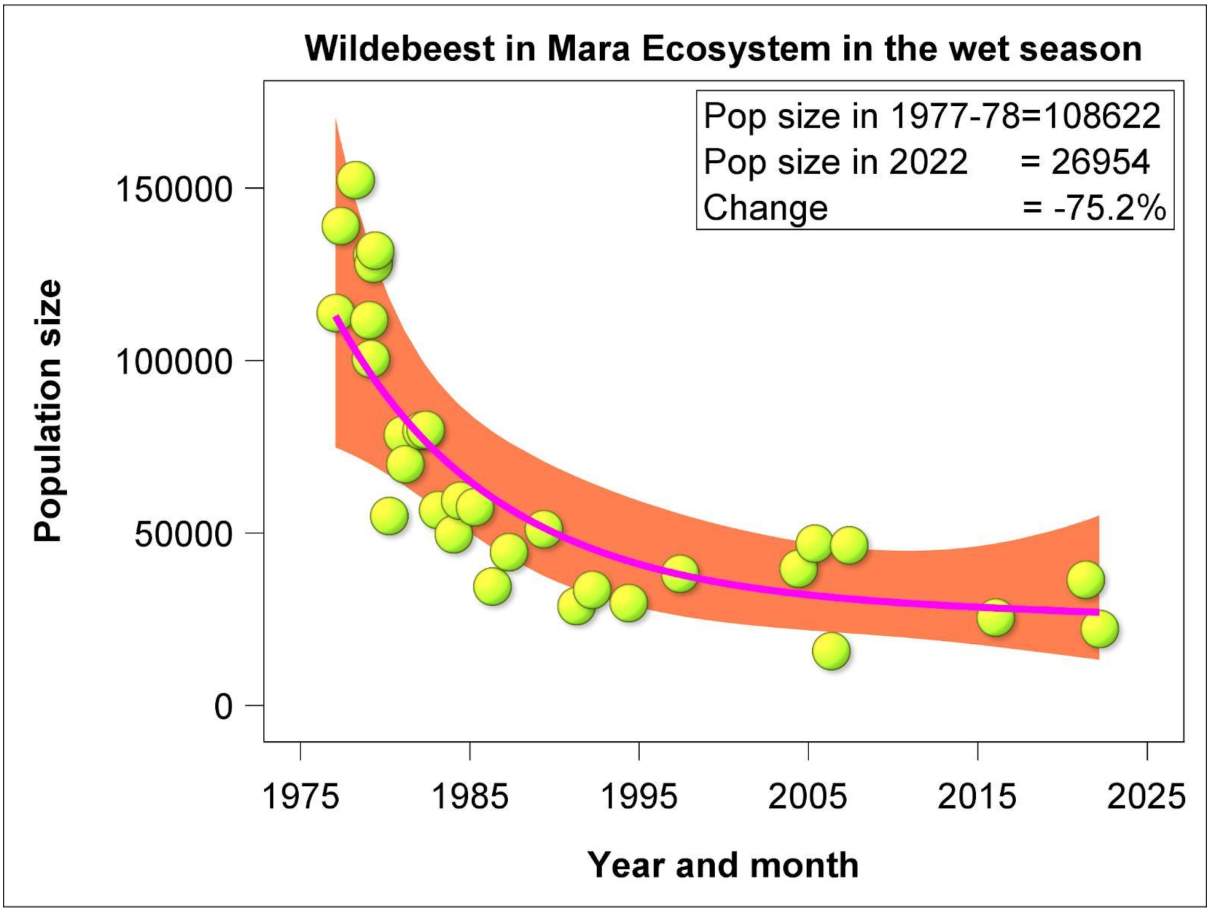

Fig. S1: Wet season wildebeest population size in the Mara ecosystem 1977/78 until 2022. (Ojwang, G. O. et al. ‘unpublished data’)

Table S1

Table S1: Habitat suitability model (after Stabach et al. (2016)) explanatory variables with data sources and references to previous use in literature

Variable	Source	Details	Literature
NDVI	Didan, K. (2015). MOD13Q1 MODIS/Terra Vegetation Indices 16-Day L3 Global 250m SIN Grid V006. NASA EOSDIS Land Processes DAAC. Accessed 2022-05-25 from https://doi.org/10.5067/MODIS/MOD13Q1.006.	Value: 16-day mean Resolution: 250 m	(Boone et al., 2006)
ΔNDVI	Same as above	Difference between current and previous mean 16-day NDVI<br>Resolution: 250 m	(Boone et al., 2006)
TWI	Calculated from DEM using Slope, Catchment Area and Topographic Wetness Index tools in QGIS v. 3.16. (QGIS Development Team, 2022)<br>DEM Source:<br>Regional Centre For Mapping Of Resources For Development<br>rcmrd.africageoportal.com	DEM (SRTM) Resolution: 30 m Catchment area method: Multiple Flow Direction	(Berry, 1997)<br>(Sørensen et al., 2006)<br>(Hopcraft et al., 2014)

		TWI method: TOPMODEL	
Anthropogenic footprint	Digitised distance to anthropogenic structures from satellite imagery (ESRI 2011 Satellite Image Basemaps and GoogleEarth 2013) by Stabach (2015) and weighted by population density based on LandScan 2008 High Resolution Global Population Data Set.	Digitised on grid at scale 1:7000	(Hopcraft et al., 2014; Stabach, 2015)
Distance to woody vegetation	FAO (2022). Thematic Woody Aggregation for Kenya – AFRICOVER. Accessed 2022-05-25 from data.apps.fao.org	Calculated distance to woodland, tree and shrub cover over 40%<br>Resolution: 50 m	(Hopcraft et al., 2005)
Distance to rivers	Imagery from WRI (wri.org) Permanent and non-permanent rivers in Kenya. Edited by Stabach (2015)	Only perennial rivers<br>Resolution: 50 m	(Hopcraft et al., 2005)
Distance to primary and secondary roads	Digitised from satellite imagery (ESRI 2011 Satellite Imagery Basemaps and GoogleEarth 2013) by Stabach (2015)	Digitised on grid at scale 1:7000	(Stabach, 2015)

		Resolution:<br><br>50 m	

* NDVI = Normalised Difference Vegetation Index; TWI = Topographic Wetness Index

References

Berry, H. H. (1997). Aspects of wildebeest Connochaetes taurinus ecology in the Etosha National Park-a synthesis for future management. Madoqua, 1, 137–145. https://doi.org/10.10520/AJA10115498_465

Boone, R. B., Thirgood, S. J., & Hopcraft, J. G. C. (2006). Serengeti wildebeest migratory patterns modeled from rainfall and new vegetation growth. *Ecology*, *87*(8), 1987–1994.

Hopcraft, G. J. C., Sinclair, A. R. E., & Packer, C. (2005). Planning for success: Serengeti lions seek prey accessibility rather than abundance. Journal of Animal Ecology, 74(3), 559–566. https://doi.org/10.1111/J.1365-2656.2005.00955.X

Hopcraft, J. G. C., Morales, J. M., Beyer, H. L., Borner, M., Mwangomo, E., Sinclair, A. R. E., Olff, H., & Haydon, D. T. (2014). Competition, predation, and migration: individual choice patterns of Serengeti migrants captured by hierarchical models. Ecological Monographs, 84(3), 355–372. https://doi.org/10.1890/13-1446.1

Sørensen, R., Zinko, U., & Seibert, J. (2006). On the calculation of the topographic wetness index: evaluation of different methods based on field observations. Hydrology and Earth System Sciences, 10, 101–112. www.copernicus.org/EGU/hess/hess/10/101/

Stabach, J. A. (2015). *Movement, Resource Selection, and the Physiological Stress Response of White-bearded Wildebeest*. Colorado State University.

QGIS Development Team. (2022). *QGIS Geographic Information System*. Open Source Geospatial Foundation Project http://qgis.osgeo.org.

Table S2

Table S2 Variance inflation of covariates in habitat selection model

Covariate	VIF
NDVI	1.01
ΔNDVI	1.02
TWI	1.14
Distance to woody vegetation	2.36
Distance to rivers	1.62
Distance to primary road	2.02
Distance to secondary road	1.71
Anthropogenic footprint	1.15
**Quadratic terms**	
(Distance to woody vegetation)2	1.72
(Distance to rivers)2	1.52
(Distance to primary road)2	1.19
(Distance to secondary road)2	1.74

*all variables were standardised

Table S3

Table S3 Pearson's Correlation Coefficients between covariates of habitat selection model. For correlation of quadratic terms see Table S4 below

	**NDVI**	**ΔNDVI**	**TWI**	**Distance to woody vegetation**	**Distance to rivers**	**Distance to primary road**	**Distance to secondary road**	**Anthropogenic footprint**
**NDVI**	1	-0.01	0	-0.05	-0.03	-0.01	-0.01	0.01
**ΔNDVI**	-0.01	1	0.01	-0.05	0.02	0.02	-0.07	0
**TWI**	0	0.01	1	-0.07	-0.25	-0.07	-0.04	0.03
**Distance to woody vegetation**	-0.05	-0.05	-0.07	1	0.07	0.54	-0.08	-0.27
**Distance to rivers**	-0.03	0.02	-0.25	0.07	1	0.08	0.02	-0.04
**Distance to primary road**	-0.01	0.02	-0.07	0.54	0.08	1	0.02	-0.31
**Distance to secondary road**	-0.01	-0.07	-0.04	-0.08	0.02	0.02	1	0.03
**Anthropogenic footprint**	0.01	0	0.03	-0.27	-0.04	-0.31	0.03	1
**(Distance to woody vegetation)2**	-0.04	-0.06	-0.08	0.36	0.15	0.23	-0.13	0.01
**(Distance to rivers)2**	-0.02	0.02	-0.01	-0.08	0.62	-0.01	0.06	0.12
**(Distance to primary road)2**	-0.01	-0.01	0.03	-0.08	0.1	-0.04	0.07	0.02
**(Distance to secondary road)2**	0.02	-0.04	-0.07	-0.06	-0.02	0.08	0.67	0

*all covariates were standardised

Table S4

Table S4 Pearson's Correlation Coefficients between covariates with quadratic term of habitat selection model

	(Distance to woody vegetation)2	(Distance to rivers)2	(Distance to primary road)2	(Distance to secondary road)2
NDVI	-0.04	-0.02	-0.01	0.02
ΔNDVI	-0.06	0.02	-0.01	-0.04
TWI	-0.08	-0.01	0.03	-0.07
Distance to woody vegetation	0.36	-0.08	-0.08	-0.06
Distance to rivers	0.15	0.62	0.1	-0.02
Distance to primary road	0.23	-0.01	-0.04	0.08
Distance to secondary road	-0.13	0.06	0.07	0.67
Anthropogenic footprint	0.01	0.12	0.02	0
(Distance to woody vegetation)2	1	0.11	0.2	-0.17
(Distance to rivers)2	0.11	1	0.06	0.03
(Distance to primary road)2	0.2	0.06	1	0.03
(Distance to secondary road)2	-0.17	0.03	0.03	1

*all covariates were standardised

Section S1: Validation

Validation approach

The peri-fencing movement data (2017-2021) were used in validation. In addition to occurrences 50 pseudo-absences were randomly distributed around each presence within a buffer of the maximum observed step length of each animal (following Stabach et al. 2016). We tested whether the models predicted the validation data locations well, by looking for a significant effect of presence or pseudo-absence on the modelled metric. We validated predictions of historic habitat suitability, historic connectivity levels, fenced connectivity levels, and the change between historic and present connectivity levels each at the regional scale across the entire Mara and at a finer local scale covering only the restoration area of interest. This was achieved by cropping the model rasters and the wildebeest movement data by the restoration area of interest (covering only the three corridors between Naboisho and Maji Moto) shown in Figure 5c. Significance was assessed by whether the 95% confidence intervals did not overlap with 0. Comparisons between presences and pseudo-absences were made using a generalised linear mixed effects model framework (GLMM) with appropriate families and link functions, after confirming compliance with model assumptions using the DHARMa package (Hartig & Lohse, 2022).

At the regional scale suitability was assumed to come from a beta distribution and hence modelled in glmmTMB (Brookes et al., 2017), while connectivity (cumulative current modelled in Circuitscape) and the change in connectivity levels were roughly normally distributed (current was log transformed) and hence models were created in lme4 (Bates et al. 2015). At the local restoration scale suitability was logit transformed and assumed to come from a normal distribution, pre-fence connectivity was log transformed and assumed to come from a normal distribution and connectivity change was normally distributed hence all were modelled in lme4 (Bates et al. 2015). All models included a random effect for animal ID (AID), to account for individual differences. All validation was performed in R (v. 4.2.0; R Core Team, 2022) using the lme4 (Bates et al. 2015) and glmmTMB (Brookes et al., 2017) packages as well as the DHARMa package (Hartig & Lohse, 2022).

General model structure: $Y \sim PA\beta + AIDu + \varepsilon$

Where Y is the response validated, PA is the predictor (presence or pseudo-absence of 2017-2021 movement data) and β its coefficient of the validated metric, u is the coefficient of the random effect included for animal ID, and ε the unexplained variation.

References

Bates, D., Mächler, M., Bolker, B., Walker, S. (2015). Fitting Linear Mixed-Effects Models Using lme4. *Journal of Statistical Software*, 67(1), 1–48. doi:10.18637/jss.v067.i01

Brooks, M. E., Kristensen, K., van Benthem, K. J., Magnusson, A., Berg, C. W., Nielsen, A., Skaug, H. J., Mächler, M., Bolker, B.M. (2017) glmmTMB Balances Speed and Flexibility Among Packages for Zero-inflated Generalized Linear Mixed Modeling PDF download. *The R Journal*, 9(2), 378-400.

R Core Team. (2022). *R: A language and environment for statistical computing* (4.2.0). R Foundation for Statistical Computing.

Stabach, J. A., Wittemyer, G., Boone, R. B., Reid, R. S., & Worden, J. S. (2016). Variation in habitat selection by white-bearded wildebeest across different degrees of human disturbance. *Ecosphere*, *7*(8), e01428. https://doi.org/10.1002/ECS2.1428

Table S5

Table S5: Coefficient estimates with confidence intervals of wildebeest habitat selection model based on 2010-2013 movement data collected by Stabach et al. (2016) compared with Stabach's wet and dry season model coefficients

Parameter	Our model	Stabach WET	Stabach DRY	Coefficient match
Anthropogenic footprint	-0.58 (0.01)	−0.47 (0.02)	−0.80 (0.03)	~WET
NDVI	-0.34 (0.00)	−0.11 (0.01)	−0.32 (0.02)	DRY
ΔNDVI	0.16 (0.00)	−0.11 (0.01)	−0.12 (0.01)	WET + DRY
TWI	-0.18 (0.00)	0.02 (0.01)	0.03 (0.01)	-
Distance to woody vegetation	0.42 (0.01)	0.61 (0.03)	0.68 (0.05)	~WET+DRY
(Distance to woody vegetation)^2	-0.15 (0.00)	−0.65 (0.03)	−0.98 (0.05)	Direction
Distance to rivers	-0.02 (0.00)	0.19 (0.02)	0.50 (0.06)	-
(Distance to rivers)^2	-0.04 (0.00)	−0.28 (0.05)	−1.57 (0.17)	Direction
Distance to primary road	-0.43 (0.01)	0.02 (0.01)†	0.17 (0.04)	-
(Distance to primary road)^2	-0.18 (0.00)	−0.00 (0.01)†	−0.17 (0.04)	DRY
Distance to secondary road	-0.35 (0.00)	−0.52 (0.02)	−0.74 (0.03)	~WET
(Distance to secondary road)^2	0.18 (0.00)	0.41 (0.02)	0.59 (0.03)	Direction

Standard errors in parentheses. All coefficients shown, except those marked with †, are significant at p = 0.001. Symbol † marks coefficients with confidence intervals that overlap 0. Distances measured in kilometres. All coefficients were standardised. Coefficient match of our model is indicated by matching either the WET season or the DRY season model, both (WET+DRY) or neither ("-"). "Direction" indicates that only the direction but not the size of effect matches.

Figure S2

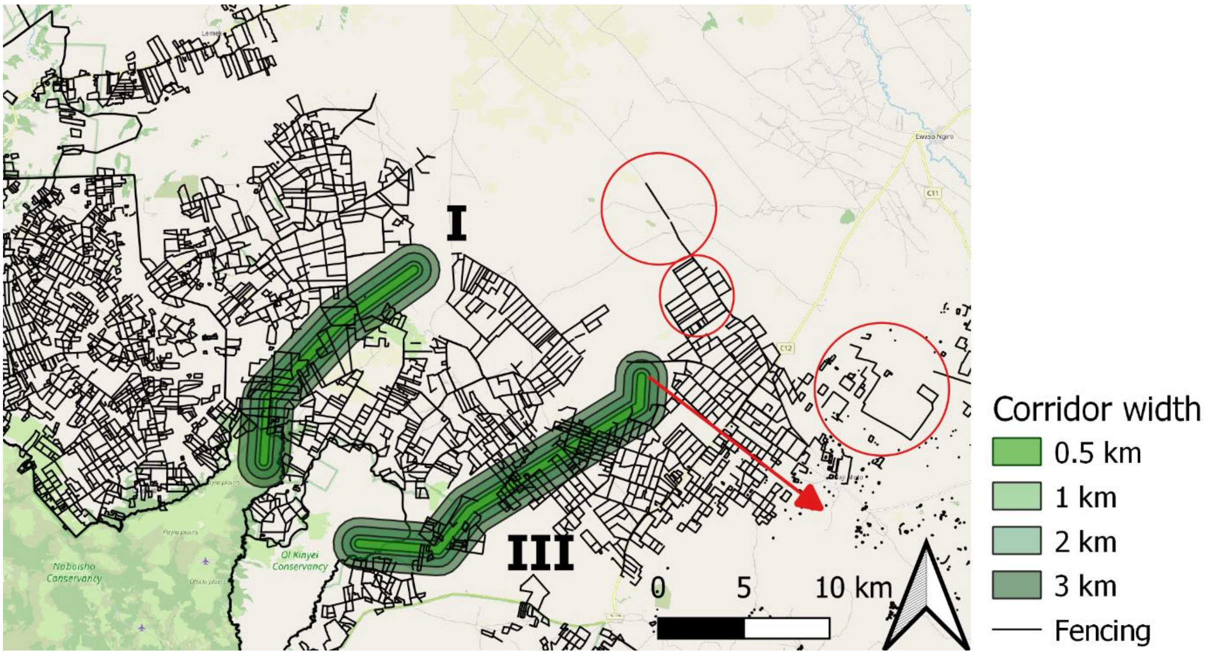

Fig. S2: Additional features to remediate if Corridor III were chosen (encircled, red) and placement of suggested corridors within the context of the wider landscape. Corridor I leads into much more open areas, than Corridor III which terminates near a densely fenced expanding cluster. Red arrow indicating potential further corridor to avoid long circumventing movements.
